# Combined Office-based Vergence Therapy and Home Therapy System for Convergence Insufficiency in Egyptian Children

**DOI:** 10.2174/1874364101812010012

**Published:** 2018-02-28

**Authors:** Tarek Nehad, Tamer Salem, Mohamed Nagy Elmohamady

**Affiliations:** Ophthalmology Department, Faculty of Medicine, Benha University, Benha, Arab Republic of Egypt

**Keywords:** Convergence insufficiency, Orthoptic Therapy, Home therapy system, Office based vision therapy, Vision therapy, Binocular vision

## Abstract

**Background:**

Convergence Insufficiency (CI) is a common binocular vision disorder characterized by exophoria more at near than at far, a receded Near Point of Convergence (NPC), and decreased Positive Fusional Vergence (PFV) at near. This disorder is often associated with several symptoms that may disturb the person’s quality of life. Therefore, diagnosis and treatment of CI is a vital issue.

**Objectives:**

To compare therapeutic yield of Office Based Vision Therapy (OBVT) and combined OBVT with Home Therapy System (HTS) in patients with CI.

**Methods:**

The study included 102 patients with age range of 7-13 years. All patients underwent Convergence Insufficiency Symptom Survey (CISS) scoring, estimation of Near Point of Convergence (NPC) and determination of Positive Fusional Vergence at near (PFV) using Sheard’s criterion. Patients were randomly allocated in two groups: Group I: received Office-based Vision Therapy (OBVT) and Group II: received OBVT with home reinforcement using the Home Therapy System (HTS). At the end of 12^th^ week of therapy; outcome was determined as *Successful* (all the following: CISS score of <16, NPC <6 cm and PFV >15Δ), Improved (CISS score of <16 or a 10 points-decrease and one of the following: NPC <6cm or *improved* by >4 cm, PFV >15Δ or increased by > 10Δ), *Insufficient response* (NPC <6cm or improved by >4 cm, PFV >15Δ or increased by > 10Δ) and non-responders.

**Results:**

At the end of the 12^th^ week of therapy, the applied therapeutic polices were successful in 48 patients (47.1%), the symptoms were improved in 30 patients (29.4%), improvement was insufficient in 13 patients (12.7%) and 11 patients (10.8%) were considered as non-responders. There was significantly higher frequency of patients with improved outcome in group II (86%) compared to group I (69.2%).

**Conclusion:**

OBVT with home supplement using HTS provided a high success rate, and it seems to be superior to OBVT alone in treatment of children with convergence insufficiency after 12-week course of therapy.

## 1 INTRODUCTION

Convergence Insufficiency (CI) is a common binocular vision disorder characterized by exophoria more at near than at far, a receded Near Point of Convergence (NPC), low Accommodative Convergence/ Accommodation (AC/A) ratio, and decreased Positive Fusional Vergence (PFV) at near. In studies that utilized standardized definitions of CI, researchers have informed an occurrence of 4.2% to 6% in school and clinic settings [1-3]. Frequently, CI causes symptoms such as double vision, sore eyes, blurred vision, sleepiness, difficulty concentrating, movement of print, headaches during near work and lessened concentration after short periods of carrying out near activities such as computer viewing. Thus, CI may undesirably influence health-related quality of life, potentially interfering with reading and near work done for school, work, and/or leisure [1, 4, 5]. The association between CI and Attention Deficit Hyperactivity disorder (ADH) has been documented in children [6]. Therefore, detecting and managing CI is an important issue in the field of binocular vision [7] ^.^

Considerable uncertainty and disagreement has existed regarding the management of convergence insufficiency [4, 8, 9]. There a clinical evidence for the efficiency of vision therapy for CI [10,11]. Insufficient evidence exists on the top therapeutic choices for treatment of the other non-strabismic binocular anomalies [12]. Therefore, the current study was designed to compare therapeutic yield of Office-based Vision Therapy (OBVT) and combined OBVT with Home Therapy System (HTS) in patients with convergence insufficiency.

## 2 MATERIALS & METHODS

The current comparative prospective study was conducted at Benha University hospital from May 2013 till April 2017. The study protocol was approved by the Local Ethical Committee and parents’ fully informed written consent was obtained according to the declaration of Helsinki. Inclusion criteria were an age of less than 16 years, exodeviation at near for at least 4Δ more than at far, a NPC break of ≥6 cm and insufficient PFV at near defined as failing Sheard’s criterion [PFV <twice the near phoria] or minimum PFV of ≤15Δ base-out blur or break, and a CI Symptom Survey (CISS) score of ≥16. CISS includes 15 questions, the patient answers each question with one of the following: never, infrequently, sometimes, often, and always. There is an algorithm to calculate the score and this was repeated after treatment to detect the level of improvement in symptoms. The evaluation before treatment and during follow up visits was done by one of the ophthalmologists authoring this study [1]. The exclusion criteria were constant strabismus, past-history of cover therapy, past-history of strabismus surgery or lack of facilities for home computer.

A total of 128 patients with CI (78 patient from Benha University clinic and 50 cases referred from nearby eye centers) were enrolled in the study but 15 cases refused the consent and 11 cases lost follow up.

### 2.1 Therapy Protocol Included the Following Forms

#### 2.1.1 Home Therapy System (HTS) (www.visiontherapysolutions.net) Computer Software

Patients, using this program, performed fusional vergence therapy actions including vergence base-in, vergence base-out, auto-slide vergence, and jump ductions vergence programs by means of random dot stereopsis targets. The HTS software program was used for 20 minutes daily for 6 days/week.

##### 2.1.1.1 Office-Based Vision Therapy (OBVT)

Patients had a weekly 60-minute in-office therapy. At each office-based therapy session, the patient completed 4-5 procedures, such as the brock string, binocular accommodative rock – flipper, stereograms, vectograms, tranaglyphs and stereoscopes under supervision from one of the authors.

## 3 PATIENTS WERE RANDOMLY ALLOCATED IN TWO GROUP

**Group I**: included patients assigned for OBVT.

**Group II**: included patients assigned for OBVT with reinforcement using the HTS.

## 4 FOLLOW-UP AND OUTCOMES

Follow-up visits were conducted after 4, 8 and 12 weeks of treatment. The primary outcome assessment was made at the 12^th^ week of treatment. At follow-up visits, a “*successful*” outcome was defined as a score of <16 on the CISS, a normal NPC (<6 cm), and normal PFV (>15Δand passing Sheard’s criterion). “*Improved*” was defined as a score of <16 or a 10 point decline in the CISS score, and at least one of the following: normal NPC, an improvement in NPC of >4 cm, normal PFV or an rise in PFV of > 10Δ. *Insufficient responder* was defined as patients had a CISS score of >16 but had a 10 point reduction in the CISS score and either normal NPC or improved by >4 cm, normal PVF or upsurge in PFV of >10Δ. Patients who did not fulfill any of these criteria were considered “*non*-*responders*” [1].

## 5 STATISTICAL ANALYSIS

Data were presented as means, medians, ranges, numbers and ratios. The Kolmogorov-Smirnov test was employed to judge the normal distribution of the data. Results were evaluated using Wilcoxon's ranked test for unrelated data (Z-test) and Chi-square test (X^2^ test) for numerical data. Statistical analysis was shown using the SPSS (Version 15, 2006). *P* value <0.05 was considered statistically significant.

## 6 RESULTS

The study included 102 patients; 47 males and 55 females with mean age of 9.1; range 7-13 years. There was a non-significant (p>0.05) difference between the groups as for age, gender, baseline examination data (Table **1**).

Response to treatment in both groups after 12 weeks was evaluated by changes in CISS, NPC and finally PFV (Table **2**). In group I, 26 cases had CISS score of > 16 from whom 13 cases improved > 10 points, while in group II, 15 cases had CISS score from whom 9 cases showed an improvement > 10 points. NPC improved > 4 cm in 26 cases in group I and in 45 cases in group II. PFV improved > 10 Δ in 26 cases in group I and in 36 cases in group II.

Collectively, at the end of the 12^th^ week of therapy, the applied therapeutic polices were successful in 19 patients in group I and in 25 patients in group II, the symptoms were improved in 17 patients in group I and in 18 patients in group II, improvement was insufficient in 8 patients in group I and in 4 patients in group II. While, 8 patients in group I and 3 patients in group II were considered as non-responders. There was a significantly higher frequency of patients had successful or improved outcome in group II (50% successful and 36% improved) compared to group I (36.5% successful and 32.7% improved) (Table **3**).

Statistically significant differences between baseline and post-treatment values of CISS, NPC and PFV were found. Baseline mean CISS was 27.9 and improved to 15.9 after treatment in group I and was 27.7 and changed to 14.6±4 in group II. As for NPC, it decreased from 10 cm and 9.5 cm, before treatment, to 5.1 cm and 4.9 cm, after treatment, in groups I and II, respectively. PFV increased from 4.2Δ to 14.5 Δ in group I, and from 4.1 Δ to 14.9 Δ in group II (Table **4**).

## 7 DISCUSSION

The two applied therapeutic policies were proved effective in managing CI as evidenced by the high portion of patients had successful or improved outcome in the two studied groups, these results are satisfactory but not complete. In this study, we evaluated cases after 12 weeks as suggested by The Convergence Insufficiency Treatment Trial Study Group (CITT) [12], but the cases with insufficient or no improvement may need a longer period of treatment as proposed by Nawrot P *et al*. They evaluated extended (24 weeks) home-based vision therapy for CI in young adults, their results showed that extending the duration of treatment might be helpful in adults with CI [13]. We chose to follow the CITT because we have almost the same age group.

In our study, OBVT proved to be effective in treating CI as 36.5% of cases showed successful outcome and 32.7% showed improvement. These results are in line with the results of CITT which reported that most children aged 9 to 17 years who were asymptomatic after a 12-week treatment program of Office-based Vergence/Accommodative Therapy (OBVAT) for CI maintained their improvements for a minimum 1 year after discontinuing treatment [12]. Borsting *et al*. found a successful or improved result after CI treatment was accompanied by a drop in the incidence of adverse academic performances and parental anxiety related to reading and school work as conveyed by parents [14].

Shin *et al*. assessed the effectiveness of in-clinic Vision Therapy (VT) and found that symptom scores and clinical measures of the treatment showed significant differences after accomplishment of 12 weeks of treatment, while no significant alteration of either symptoms or signs were evident for the control group, and one year follow-up examination revealed that most children maintained the improved symptom and clinical measures after VT supporting the notion that VT is a successful method of treating CI [15].

HTS has been evaluated separately in some studies where Brautaset and Jennings evaluated the effect of home orthoptic treatment on the AC/A in subjects with CI. They found no statistically significant change in the AC/A (P>0.05) after orthoptic treatment [16] ^.^

Cooper & Feldman found automated vision therapy delivered by the HTS system enhanced convergence amplitudes with a concomitant lessening in symptoms and recommended its usage for those patients with symptoms related with a vergence anomaly especially when in-office vision therapy is not available [17]. Serna *et al*. tried to gauge the efficacy of a home-based computer orthoptic program to treat symptomatic CI and found that post-treatment mean NPC improved from 24.2 to 5.6 cm with 92.8% of patients attained an NPC of ≤6 cm, and PFC improved in these patients (92.8%) and a whole of 27 patients (64.2%) reported to resolve the symptoms after treatment [18] ^.^

Kim & Chun assessed the usefulness of home-based pencil pushups (HBPP) therapy for patients with symptomatic CI and after 12 weeks of HBPP therapy found the mean deviation angle of exophoria was diminished to orthophoric at distant and 4 PD at near, the mean value of NPC was lessened to 14.4 cm and settled that 12-weeks of HBPP therapy seems to be an easy, cost-free and effective therapy for subjects with symptomatic CI [19].

Combined OBVT and HTS allowed significantly higher frequency of success (50%) and improvement (36%) in comparison to OBVT alone (36.5% success and 32.7% improvement). These findings go in hand with Scheiman *et al*. who found that the rate of advancement was more for clinical signs (NPC and PFV) than for symptoms in children experiencing treatment for CI and that OBVAT with home fortification resulted in a more rapid improvement in symptoms, NPC and PFV, and a greater section of patients attaining pre-determined criteria of success when compared with home-based computer vergence/accommodative therapy and pencil pushups, or office-based placebo therapy with home augmentation [20] ^.^

## CONCLUSION

It could be concluded that office-based vergence therapy with home supplement using the home therapy system provided success rate of about 84% in children with CI after 12-week course of therapy. Eye care specialists who do not currently offer this treatment may consider referring these patients to a specialized center which offers this treatment or consider expanding the treatment choices accessible within their practice to manage this condition.

## Figures and Tables

**Table 1:** Patients’ enrolment criteria.

–		Group I	Group II
Age (years)		9.1±1.6	9.3±1.2
Gender	Males	23 (44.2%)	24 (48%)
	Females	29 (55.8%)	26 (52%)
Glass Wearer	Yes	16 (30.8%)	17 (34%)
	No	36 (69.2%)	33 (66%)
CISS Score		27.9±4.6	27.7±3.9
NCP (cm)		10±2.6	9.5±3.1
PFV (Δ)		11.1±1.7	11±1.7

Data are presented as mean ±SD & numbers; percentages are in parenthesis; CISS: Convergence insufficiency Symptom Survey score; NPC: Near convergence point; PFV: positive fusional vergence.

**Table 2:** CISS score, NPC and PFV data recorded at the end of the 12^th^ week of therapy.

–	–	Group I	Group II	
CISS<16	Improvement ≥10	17 (32.7%)	22 (44%)	
	Improvement <10	9 (17.3%)	13 (26%)	
CISS>16	Improvement ≥10	13 (25%)	9 (18%)	
	Improvement <10	13 (25%)	6 (12%)	
Statistical Significance from Baseline		X^2^=3.19, p<0.05		
NPC<6 & Improvement ≥4		24 (46.2%)	38 (76%)	
NPC<6 & Improvement <4		16 (30.7%)	5 (10%)	
NPC>6 & Improvement ≥4		12 (23.1%)	7 (14%)	
Statistical Significance from Baseline		X^2^=3.257, p<0.05		
Normal PFV & Improvement >10Δ		22 (42.3%)		32 (64%)
Normal PFV & Improvement ≤10Δ		16 (30.8%)		14 (28%)
Insufficient PFV & Improvement >10Δ		14 (26.9%)		4 (8%)
Statistical Significance		X^2^=5.195, p<0.05		

Data are presented as mean ±SD & numbers; percentages are in parenthesis; CISS: Convergence insufficiency Symptom Survey score; NPC: Near convergence point; PFV: positive fusional vergence

**Table 3:** The overall response at the end of the 12^th^ week of therapy.

–	Group I	Group II
Success	19 (36.5%)	25 (50%)
Improved	17 (32.7%)	18 (36%)
Insufficient	8 (15.4%)	4 (8%)
Non-Responders	8 (15.4%)	3 (6%)
Statistical Significance	X^2^=3.415, p<0.05	

Data are presented as mean ±SD & numbers; percentages are in parenthesis.

**Table 4:** Differences between baseline and post-treatment values of CISS, NPC and PFV.

–		Group I	Group II
Mean CISS Score	Baseline	27.9±4.6	27.7±3.9
	At 12^th^ Week	15.9±3.7	14.6±4
	Statistical Significance	**Z=1.011, p**<**0.05**	
Mean NPC	Baseline	10±2.6	9.5±3.1
	At 12^th^ Week	5.1±1.6	4.9±1.6
	Statistical Significance	**Z=0.754, p**<**0.05**	
Mean PFV	Baseline	4.2±1.5	4.1±1.4
	At 12^th^ Week	14.5±3.1	14.9±1.9
	Statistical Significance	**Z=1.335, p**<**0.05**	

Data are presented as mean ±SD; **CISS**: Convergence insufficiency Symptom Survey score; **NPC**: Near convergence point in centimeters; **PFV**: positive fusional vergence in prism diopters.

## LIST OF ABBREVIATIONS

• **AC/A**: = Accommodative Convergence/ Accommodation.
• **CI**: = Convergence Insufficiency.
• **CISS**: = Convergence Insufficiency Symptoms Survey.
• **CITT**: = Convergence Insufficiency Treatment Trial.
• **HTS**: = Home Therapy System.
• **NPC**: = Near Point of Convergence.
• **OBVT**: = Office Based Vision Therapy.
• **PFV**: = Positive Fusional Vergence.
• **VT**: = Vision Therapy.

## References

[1] Scheiman M., Mitchell G.L., Cotter S., Convergence insufficiency treatment trial study group (2008). Randomized clinical trial of treatments for symptomatic convergence insufficiency in children.. Arch. Ophthalmol..

[2] Cooper J, Jamal N. (2012). Convergence insufficiency: A major review.. Optometry.

[3] Scheiman M., Rouse M., Kulp M.T., Cotter S., Hertle R., Mitchell G.L. (2009). Treatment of convergence insufficiency in childhood: A current perspective.. Optom. Vis. Sci..

[4] Bade A., Boas M., Gallaway M., Mitchell G.L., Scheiman M., Kulp M.T., Cotter S.A., Rouse M., CITT study group (2013). Relationship between clinical signs and symptoms of convergence insufficiency.. Optom. Vis. Sci..

[5] Granet D.B., Gomi C.F., Ventura R., Miller-Scholte A. (2005). The relationship between convergence insufficiency and ADHD.. Strabismus.

[6] Hashemi H., Nabovati P., Khabazkhoob M., Ostadimoghaddam H., Doostdar A., Shiralivand E., Yekta A. (2017). The prevalence of convergence insufficiency in Iran: A population-based study.. Clin. Exp. Optom..

[7] Scheiman M., Mitchell G.L., Cotter S., Cooper J., Kulp M., Rouse M., Borsting E., London R., Wensveen J., Convergence insufficiency treatment trial study group (2005). A randomized clinical trial of treatments for convergence insufficiency in children.. Arch. Ophthalmol..

[8] Convergence insufficiency treatment trial study group (2008). Randomized clinical trial of treatments for symptomatic convergence insufficiency in children.. Arch. Ophthalmol..

[9] Shin H.S., Park S.C., Maples W.C. (2011). Effectiveness of vision therapy for convergence dysfunctions and long-term stability after vision therapy.. Ophthalmic Physiol. Opt..

[10] Cacho Martínez P., García Muñoz A., Ruiz-Cantero M.T. (2009). Treatment of accommodative and nonstrabismic binocular dysfunctions: A systematic review.. Optometry.

[11] McGregor M.L. (2014). Convergence insufficiency and vision therapy.. Pediatr. Clin. North Am..

[12] Convergence insufficiency treatment trial study group. Long-Term effectiveness of treatments for symptomatic convergence insufficiency in children. (2009). Optometry and vision science: official publication of the American Academy of Optometry..

[13] Nawrot P., Michalak K.P., Przekoracka-Krawczyk A. (2013). Does home-based vision therapy affect symptoms in young adults with convergence insufficiency?. Opt. Appl..

[14] Borsting E., Mitchell G.L., Kulp M.T., Scheiman M., Amster D.M., Cotter S., Coulter R.A., Fecho G., Gallaway M.F., Granet D., Hertle R., Rodena J., Yamada T., CITT study group (2012). Improvement in academic behaviors after successful treatment of convergence insufficiency.. Optom. Vis. Sci..

[15] Shin H.S., Park S.C., Park C.M. (2009). Relationship between accommodative and vergence dysfunctions and academic achievement for primary school children.. Ophthalmic Physiol. Opt..

[16] Brautaset R.L., Jennings A.J. (2006). Effects of orthoptic treatment on the CA/C and AC/A ratios in convergence insufficiency.. Invest. Ophthalmol. Vis. Sci..

[17] Cooper J., Feldman J. (2009). Reduction of symptoms in binocular anomalies using computerized home therapy-HTS.. Optometry.

[18] Serna A., Rogers D.L., McGregor M.L., Golden R.P., Bremer D.L., Rogers G.L. (2011). Treatment of symptomatic convergence insufficiency with a home-based computer orthoptic exercise program.. J. AAPOS.

[19] Kim K.M., Chun B.Y. (2011). Effectiveness of Home-based Pencil Push-ups (HBPP) for patients with symptomatic convergence insufficiency.. Korean J. Ophthalmol..

[20] Scheiman M., Kulp M.T., Cotter S., Mitchell G.L., Gallaway M., Boas M., Coulter R., Hopkins K., Tamkins S., Convergence insufficiency treatment trial study group (2010). Vision therapy/orthoptics for symptomatic convergence insufficiency in children: Treatment kinetics.. Optom. Vis. Sci..

